# Identification of Peptide Lv, a Novel Putative Neuropeptide That Regulates the Expression of L-Type Voltage-Gated Calcium Channels in Photoreceptors

**DOI:** 10.1371/journal.pone.0043091

**Published:** 2012-08-13

**Authors:** Liheng Shi, Michael L. Ko, Louise C. Abbott, Gladys Y. -P. Ko

**Affiliations:** Department of Veterinary Integrative Biosciences, College of Veterinary Medicine and Biomedical Sciences, Texas A&M University, College Station, Texas, United States of America; University of Waterloo, Canada

## Abstract

Neuropeptides are small protein-like signaling molecules with diverse roles in regulating neural functions such as sleep/wake cycles, pain modulation, synaptic plasticity, and learning and memory. Numerous drugs designed to target neuropeptides, their receptors, or relevant pathways have been developed in the past few decades. Hence, the discovery and characterization of new neuropeptides and their functions have received considerable attention from scientific research. Computational bioinformatics coupled with functional assays are powerful tools to address the difficulties in discovering new bioactive peptides. In this study, a new bioinformatic strategy was designed to screen full length human and mouse cDNA databases to search for novel peptides. One was discovered and named peptide Lv because of its ability to enhance L-type voltage-gated calcium channel (L-VGCC) currents in retinal photoreceptors. Using matrix-assisted laser desorption/ionization-time of flight mass spectrometry (MALDI-TOF MS), peptide Lv was detected in the culture media, which indicated that it was secreted from 661W cells transfected with the gene. *In vitro* treatments with either glutathione S-transferase (GST) fusion peptide Lv or synthesized peptide Lv enhanced L-VGCC channel activities in cone photoreceptors. At the molecular level, peptide Lv stimulated cAMP production, enhanced phosphorylation of extracellular signal-regulated kinase (ERK), and increased the protein expression of L-VGCCα1 subunits in cone photoreceptors. Therefore, the biological activities of peptide Lv may be very important in the modulation of L-VGCC dependent neural plasticity.

## Introduction

Neuropeptides act like peptidic hormones or neurotransmitters and play diverse roles in regulating neural functions [1], [2], [3], [4]. Numerous drugs designed to target neuropeptides, their receptors, or relevant pathways have been developed in the past few decades [1], and the discovery and characterization of new neuropeptides and their functions have received considerable attention from both scientific research and clinical practice. Identification of new bioactive peptides is challenging because they are short in length, have limited information on their core sequences and structures, and have highly diverse physiological functions. Historically, biochemical purification coupled with functional assays was the major method for discovering neuropeptides. However, advancements in computational bioinformatics with growing DNA and protein databases have successfully predicted secretory peptides in recent years [5], [6], [7]. Small peptide hormones and neurotransmitters share some common features, including a signal peptide sequence and pre-hormone cleavage sites [8]. The Hidden Markov model, a bioinformatic model used for domain predictions, has proven successful in identifying novel peptides from the human proteome. It includes the common features of small peptides mentioned above [9]. Therefore, bioinformatic approaches coupled with activity assays have become a powerful tool to address the difficulties in discovering new bioactive neuropeptides.

Our goal was to develop a new bioinformatic strategy for discovery of novel bioactive peptides that might have important roles in regulating neural excitability and plasticity. In this study, we present a newly designed bioinformatic strategy modified from the Hidden Markov model to screen full length cDNA databases in search of novel secretory peptides. Our improved screening protocol included more propeptide convertase cutting motifs, as well as transmembrane domains in propeptide candidates, since several peptides or secretory proteins are derived from precursors with transmembrane domains [10], [11], [12]. Concurrently, several activity assays were employed to examine the bioactivities of the candidate peptide, including biochemical and electrophysiological assays.

Here, we focused on a novel peptide that we discovered through the screening process described above. The amino acid sequence of this novel peptide is highly conserved across species and is widely expressed in various tissues and the central nervous system, including the retinal photoreceptor layer, hippocampus, olfactory bulb, and cerebellum. Using patch-clamp electrophysiological recordings, we found that this peptide enhanced L-type voltage-gated calcium channel (L-VGCC) currents in photoreceptors through increasing the mRNA and protein expression of both L-VGCCα1C and α1D subunits, the major L-VGCC pore forming subunits present in neurons and retina photoreceptors [13]. Hence, we named this peptide as “peptide Lv”. In various cell types, L-VGCCs mediate voltage-dependent, depolarization-induced Ca^2+^ influx and regulate diverse biological processes such as contraction, secretion, differentiation, synaptic plasticity, and gene expression [14], [15], [16], [17]. The L-VGCCs are also essential in gating prolonged neurotransmitter release from retina photoreceptors [18]. The L-VGCC activity can be modulated by neurotransmitters, hormones, cytokines, as well as intracellular signal transduction pathways [16], [19]. To that end, peptide Lv was able to stimulate cAMP production and activate mitogen-activated protein kinase (MAPK) signaling. Hence, we may have discovered a new bioactive peptide that can potentially modulate a variety of functions associated with L-VGCCs.

## Experimental Procedure

### In Silico Screening of Novel Peptides from Human and Mouse Full Length cDNA Database

**Figure 1:**
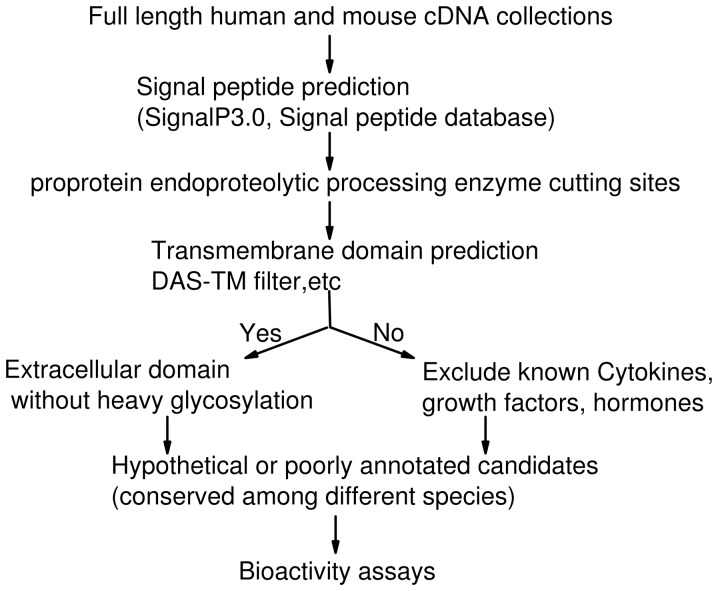
A flow chart illustration of the *in silico* computational screening strategy and procedure. Human and mouse full length protein databases were subjected to five step separation computer algorithms including N-terminal signal peptide sequence (secretion marker), propeptide convertase targeting motif, transmembrane domain, potential glycosylation modification, and sequence homology across species. Potential candidates were ranked according to the score assigned from each step (Data S1).

The purpose of the computational screening was to discover the candidate genes encoding novel bioactive peptides that have never been characterized before. Human (21,243 genes) and mouse (21,076 genes) full length cDNAs were downloaded from the database [20], [21]. The screening strategy (Fig. 1) found no new DNA sequence. Several features in the biological production of peptide hormones were taken into consideration in our screening process for candidate genes. Amino acid sequences were screened using several algorithms, including SignalP 3.0 (set as default, D-cut off at 0.45) for the prediction of signal peptides, the Dense Alignment Surface (DAS) method (>2.0 cut off) to identify transmembrane domains, and Expasy NetNGlyc 1.0 to predict N-glycosylation sites (potential threshold set as >0.6). Sequence comparisons among humans, rats, and mice (>50% homology) were carried out using the NCBI database and UCSC genomic browser. A detailed list of genes from each screening step is in Data S1. The proprotein cleavage cutting motifs mainly included dibasic amino acid sequences such as GKK/GRR, RKX, RRX, KR, RXKR, KXRR, and RFGK. We selected 5 candidate genes based on their distribution in the Expressed Sequence Tag (EST) database as potential “proproteins” and examined their bioactivities. Here, we present one of the candidate genes that encoded “peptide Lv”.

### Cloning of Mouse Peptide Lv Full Length cDNA

To clone the candidate cDNA, a commercially available kit (Qiagen, Valencia, CA) was used to isolate total RNA from an adult male C57BL6 mouse cerebral cortex. 1 µg total RNA was subjected to reverse transcription for first strand synthesis (Invitrogen, Carlsbad, CA). The full length mouse E130203B14Rik cDNA (NM_178791.4) was amplified by PCR (Invitrogen; sense primer: 5′-GAATTCATGCGGCTCCTAGCGCTGG CGGCGG-3′; antisense primer: 5′-CTCGAGCTACAGCTGGTTCTCCTCGAAGAGGA-3′). The PCR fragment was purified and inserted into a pGEM T-easy vector (Promega, Madison, WI) for sequencing. For expression in mammalian cells (the 661W cell-line, described below), the full length cDNA was inserted into a pCAGIG vector.

### Cell Culture and Transfection

Fertilized eggs (*Gallus gallus*) were obtained from the Department of Poultry Sciences, Texas A&M University (College Station, TX). Dissociated chicken cone photoreceptors from embryonic day 10 (E10) or E16 were cultured for 2 days as described previously in the presence of 20 ng/ml ciliary neurotrophic factor (CNTF, R&D Systems, Minneapolis, MN), 10% heat-inactivated horse serum (Lonza, Basel, Switzerland), and 100 u/ml penicillin/100 µg/ml streptomycin (Lonza) [20]. Cultures prepared in the presence of CNTF yield a highly enriched population (70∼80%) of cone photoreceptors [21], [22], [23]. The immortalized mouse cone photoreceptor cell line, 661W, was obtained from Dr. M. R. Al-Ubaidi (University of Oklahoma College of Medicine, Oklahoma City, OK) [24]. The 661W cells were cultured in Dulbecco’s modified Eagle medium (Lonza) containing 10% fetal bovine serum (Thermo Scientific, Rockford, IL) and 100 u/ml penicillin/100 µg/ml streptomycin (5% CO_2_, 37°C). Transfections were performed using lipofectamine 2000 transfection reagent (Invitrogen). The culture media were replaced 12 hr after transfection. The new media were collected 48 hr after transfections.

### High-performance Liquid Chromatography (HPLC) and Mass Spectrometry (MS)

Media collected from cultured murine 661W photoreceptor cells were filtered and concentrated through Amicon Ultracel-10 and Ultracel-3 centrifugal filters, respectively (Millipore, Billerica, MA). After filtration, concentrated samples were loaded onto a Dionex HPLC system (Acclaim C18 column, Ultimate 3000 variable wavelength detector; Dionex, Bannockburn, IL), and a linear gradient (10%–80% acetonitrile [ACN]/H_2_O with 0.1% trifluoroacetic acid [TFA]; 30 mins; 1 ml/min) was used for final elution. The HPLC elutate (fractions from retention time 15–25 min) was concentrated (SpeedVac, Thermo Fisher Scientific, Waltham, MA) for further determination of the molecular weights by MS. MALDI-TOF MS was performed by an ABI Voyager-DE STR (Department of Chemistry, Texas A&M University, College Station, TX). Samples were ionized by sinapic acid (1∶1 ratio).

### In Situ Hybridization

Whole brains and eyes from five adult male C57BL/6 mice were collected after perfusion with 4% paraformaldehyde in PBS and post-fixed with paraformaldehyde for another 4 hr, followed by suspension in 30% sucrose/PBS overnight. Samples were then mounted in optimum cutting temperature (O.C.T.) embedding compound (Tissue-Tek; Sakura Finetek, Torrance, CA) and 12 µm saggital sections were cut (Leica Microsystems, Buffalo Grove, IL). The biotin labeled probe was the *in vitro* transcripted antisense RNA of murine peptide Lv (Roche, Indianapolis, IN) including a 300 bp region from the ATG start code in exon 1 to the *Xho*I cutting site in exon 2. Final images were obtained by alkaline phosphatase conjugated to streptavidin and substrate BCIP (5-bromo-4-chloro-3′-indolyphosphate p-toluidine salt)/NBT (nitro-blue tetrazolium chloride; Roche). All procedures involving mice were approved by the Institutional Animal Care and Use Committee (IACUC) of Texas A&M University consistent with approved protocols described in the National Institutes of Health Guide for the Care and Use of Laboratory Animals (National Institutes of Health Publication No. 85–23, revised 1996). Mice were housed under standard temperature and humidity controlled conditions with 12∶12 hr LD cycles and fed standard laboratory chow and water ad libitum.

### Immunoblotting, Quantitative (Q)-PCR, and cAMP Immunoassay

**Figure 2:**
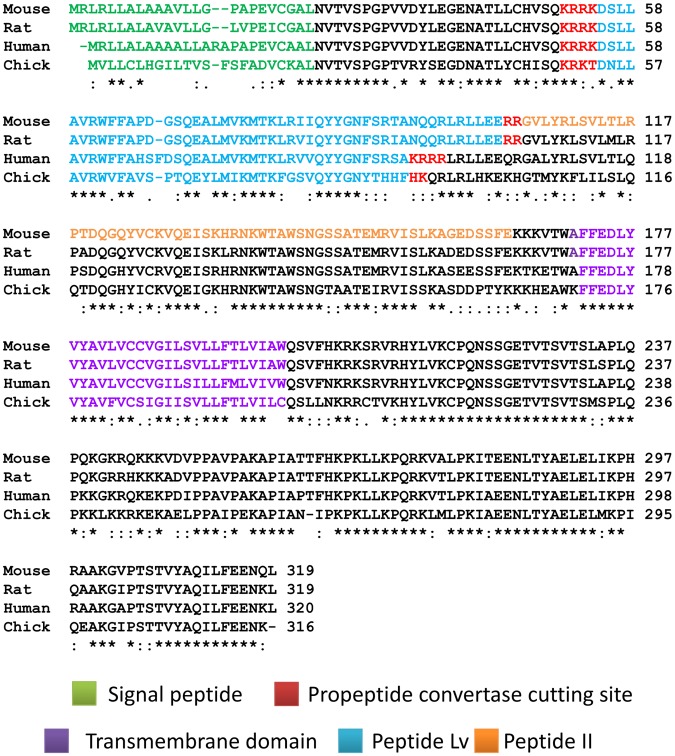
Sequence alignment of propeptide Lv among different species. Mouse, rat, human, and chicken peptide Lv proprotein were over 80% homologous. The sequence contained a signal peptide sequence (green), proprotein convertase cutting motifs (red), the main peptide Lv coding region (blue), and a transmembrane domain (purple). Peptide II (control for the electrophysiological studies) is highlighted in orange. Highly conserved identical sequences are denoted by (*), conserved substitution by (**:**), and semiconserved substitution by (**.**).

Cone photoreceptors from E10 embryos were cultured for 2 days. The cells were treated with murine GST-peptide Lv at a final concentration of 800 ng/ml or synthesized murine peptide Lv at a final concentration of 500 ng/ml for 4 hrs. Murine GST-peptide II or PBS served as control. Peptide II was derived from same encoding gene as peptide Lv as indicated in Figure 2 (orange color). The sequence of peptide II was located adjacent to the coding region of peptide Lv. Since peptide II and peptide Lv were from the same encoding gene, we selected the peptide II as an internal control to ensure that the bio-activity of peptide Lv was sequence specific. Treated or control cells were washed, lysates were obtained by suspension of cells in RIPA lysis buffer, and proteins were denatured by mixing with 2X Lamelli sample buffer for 5 min at 95°C. Primary antibodies for pan L-VGCCα1 (Chemicon/Millipore, Temecula, CA), ERK1 (sc-94, Santa Cruz Biochemicals, Santa Cruz, CA), pAKT_308_ (Cell Signaling Technology, Danvers, MA), and pERK (M9692, Sigma, St. Louis, MO) were incubated overnight at 4°C. Blots were visualized by appropriate secondary antibodies and an ECL detection system (Pierce, Rockford, IL). For some experiments, pertussis toxin (PTX; Calbiochem/EMD, San Diego, CA) was added to a final concentration of 0.5 µg/ml. For Q-PCR, peptide Lv treated or control cells were harvested, and total RNA was isolated as described above. One step RT-PCR amplification (Applied Biosystems, Foster City, CA) was used for detection of L-VGCCα1D and β-actin mRNA expression. Primers and probes for L-VGCCα1D and β-actin mRNA were listed previously [20]. For VGCCα1C, the primers and probe sequences are forward primer: 5′-TCTCGCATCTCA ATCACCTTCTTC-3′, reverse primer: 5′-GGCTCAGGAGCTTCACAAGAC-3′, and MGB-Probe: 5′-ATCACGCGGAAGAGTC-3′. For mouse GAPDH, the forward primer was 5′-CATTGTGGAAGGGCTCATGACCA-3′, and reverse primer was 5′-TGGGATGACCTT GCCCACAGCCTTG-3′. Cyclic AMP levels were detected by a commercially available kit (Arbor Assays, Ann Arbor, MI). The final cAMP concentration was normalized to protein concentration (Bradford method; Bio-rad, Hercules, CA).

### GST Fusion Protein Purification and Peptide Synthesis

The coding region of murine peptide Lv and murine peptide II (control peptide) were amplified by high fidelity PCR (Invitrogen; sense primers: 5′-ATAGAATTCGACAGCTTGCTGGCCG TGCGC-3′ [peptide Lv], 5′-ATAGAATTCGGGGTGCTGTACAGGCTGTCTG-3′ [peptide II]; antisense primers: 5′-ATACTCGAGACGCTCCTCGAGCTGGCGTAG-3′ [peptide Lv], 5′- ATACTCGAGCTCAAATGATGAAATCTTCGCC-3′ [peptide II]). PCR fragments were verified by DNA sequencing and inserted into pGEX-4T1 expression vectors (EcoRI/NotI). The plasmids were transformed into BL21 *E. coli*. The expression of fusion proteins was induced by 0.5 mM isopropyl β-D-1-thiogalactopyranoside (IPTG) and confirmed by SDS-PAGE. The GST fusion proteins were purified with a commercially available kit (Pierce, Rockford, IL). Briefly, *E. coli* cells were collected and disrupted by sonication. The supernatants were loaded onto GSH-sepharose 4B columns. The flow through and final elution from the beads were collected for SDS-PAGE. The final GST-peptide Lv product was dialyzed against PBS. Synthesized peptide Lv (over 90% pure) was purchased from Genscript (Piscataway, NJ).

### Immunoprecipitation (IP)

Ten retinas from chicken embryos (E12) were collected and lysed in 4 ml RIPA buffer containing a protease inhibitor cocktail. The cell lysate was divided into two parts (2 ml each) and incubated with either pre-bleed rabbit serum (1/500 dilution) or peptide Lv specific polyclonal antibody (1/500 dilution) for 3 hr at 4°C. The rabbit polyclonal antibody was custom-made by Biomatik (Ontario, Canada) against synthesized peptide Lv and purified by a protein A affinity column. 25 µl immobilized protein A resin (G biosciences, St. Louis, MO) were prewashed by PBS and incubated with the cell lysates for 3∼4 hr at 4°C. The resins were washed three times with 0.05% Triton X-100 in PBS and twice with PBS, then boiled with 25 µl SDS-PAGE sample buffer at 95°C for 10 min. The supernatants were loaded onto 18% SDS-PAGE gels and transferred to 0.22 µm nitrocellulose membranes (GE Healthcare life sciences, Piscataway, NJ). The membranes were probed by anti-peptide Lv antibody.

### Patch-Clamp Electrophysiology

Whole-cell voltage-clamp recordings of L-VGCCs were carried out as described previously [20]. The external solution contained the following (in mM): NaCl 110, BaCl_2_ 10, MgCl_2_ 0.4, KCl 5.3, tetraethylammonium chloride 20, HEPES 10, and glucose 5.6, pH 7.4 adjusted with NaOH. The pipette solution was (in mM): Cs acetate 135, CsCl 10, MgCl_2_ 2, CaCl_2_ 0.1, EGTA 1.1, and HEPES 10, pH 7.4 adjusted with CsOH. Recordings were made from E10+2 (E12) or E16+2 (E18) cone photoreceptors characterized morphologically as cells with elongated cell bodies with one or more oil droplets. Cells were recorded using a ramp command from −80 to 60 mV in 500 ms, and a 200 ms step command with holding potential at −65 mV and steps from −50 to 40 mV at 10 mV increments. The current density (*I*_density_) was obtained by dividing the current amplitude by the membrane capacitance. Each group contained 9–18 cells. The conductance-membrane potential curves were analyzed using the Boltzmann equation, “G/Gmax  = 1/(1+exp[Vmid-V/Ka])”. G: conductance, V: membrane voltage, Vmid: the membrane potential that elicits half maximal activation (current), Ka: the activation slope factor. Toward the end of each recording, nitrendipine (5 µM), a L-VGCC blocker, was perfused through the extracellular solution for 5 min, and the final ramp-command was recorded to ensure that each recording was the L-VGCC current.

### Statistics

All data are presented as mean ± standard error of mean (s.e.m.). The Student’s *t* test was used for statistical analysis to compare the control and treated groups. One-way ANOVA with Tukey post hoc test was used for comparing multiple groups. Throughout, *p<0.05 was regarded as significant.

## Results

### Computational Screening of Peptide Lv from the cDNA Database

We designed a new computational genomic screening strategy (Fig. 1) based on the Hidden Markov model to identify new peptide hormones. First, human and mouse full length cDNA databases [25], [26] were used as our screening source, since these cDNAs encode proteins with longer N-terminal domains that were ideal for our purposes. Second, more propeptide convertase cutting motifs including GKK/GRR, RKX, RRX, KR, RXKR, KXRR, and RFGK were included in the screening procedure. Third, some potential propeptides with unknown transmembrane domains were included as candidates, since several bioactive peptides or secretory proteins are derived from precursors with transmembrane domains, such as teneurin C-terminal-associated peptides [10], semaphoring [11], and zona proteins [12]. Hence, it is possible that propeptide candidates may contain transmembrane domains beyond the peptide’s coding region, and a peptide can be proteolytically released from a transmembrane containing proprotein. Fourth, candidates were screened for potential glycosylation modification sites through specific algorithms [27], and those sequences that undergo heavy glycosylation were eliminated. In order to specifically identify secretory peptides/proteins from the databases, several bioinformatic algorithms were developed for the identification of N-terminal signal peptides [28]. Moreover, secretory protein databases from several species are available [29]. Based on our improved screening protocol, the majority of peptide candidate genes that were identified in our screening belonged to several families of well-studied peptide hormones or small proteins.

We focused on poorly annotated candidates to decode novel bioactive peptide products with uncharacterized physiological functions. We also examined the expressed sequence tags (EST) of these candidates and chose those that were potentially expressed in neurons. Lastly, we chose candidates whose amino acid sequences were highly conserved among species (see **Data S1** for lists of candidate sequences). Here, we report the properties of the mouse E130203B14Rik gene product, which encodes a hypothetical protein with 319 amino acids that contains an N-terminal signal peptide sequence, a main peptide coding region, and a transmembrane domain predicted in the C-terminal region. The main peptide coding region from the mouse E130203B14Rik gene contains 49 amino acids, and its sequence was conserved among human, mouse, rat, and chicken (Fig. 2). We named this small protein “peptide Lv” based on its physiological activities described below.

### Peptide Lv mRNA was Widely Expressed in Mice

**Figure 3:**
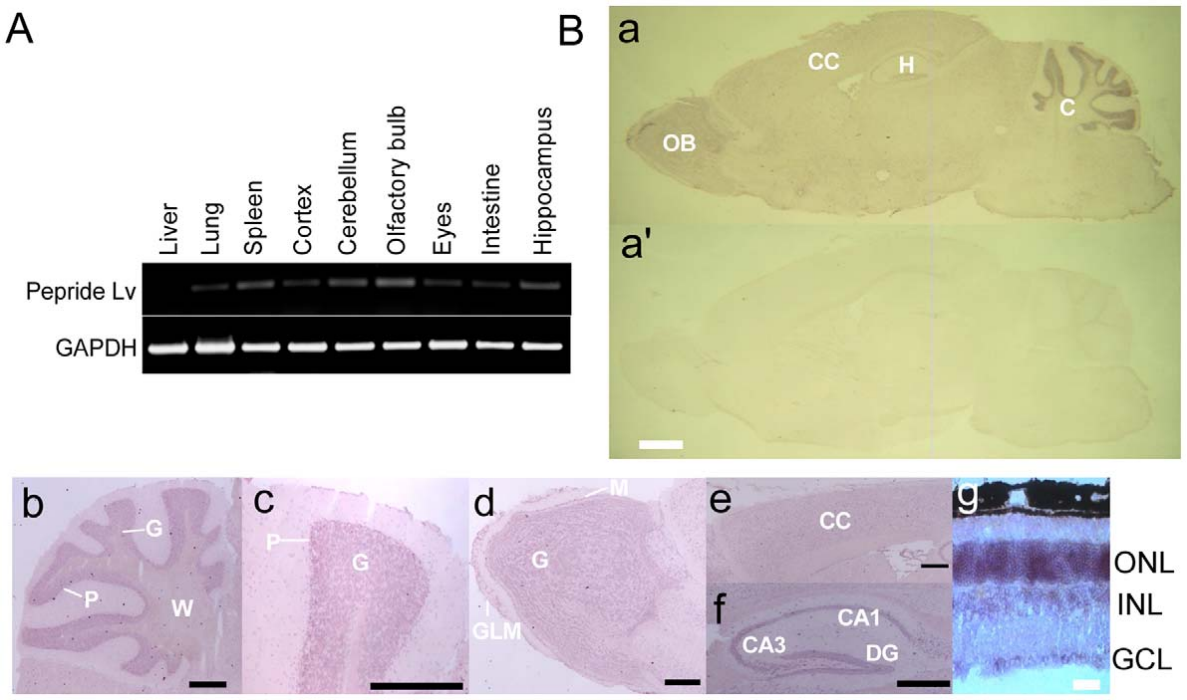
Expression of peptide Lv mRNA in mouse tissues. (A) Gene expression of peptide Lv in various mouse tissues was determined by RT-PCR. The mRNA of peptide Lv was detected in the liver, lung, spleen, intestine, eyes, cerebral cortex (cortex), cerebellum, olfactory bulb, and hippocampus, with relatively higher expression levels in the spleen, cerebellum, olfactory bulb, and hippocampus. (B) The mRNA expression of peptide Lv in the mouse brain and retina was detected by *in situ* hybridization. Adult mouse brain sagittal sections and retina sections were cut at 12 µm thickness and processed. The sense probe served as the negative control (a’). Propeptide Lv mRNA was strongly expressed in the cerebellum (C), olfactory bulb (OB), cerebral cortex (CC), and hippocampus (H). (b and c): cerebellum; P, Purkinje cell layer; G, granule cell layer; W, white matter. (d): olfactory bulb; GLM, glomerular layer; M, mitral cell layer; G, granule cells. (e): cerebral cortex. (f) hippocampus; CA, cornu ammonis; DG, dentate gyrus. (g) In the mouse retina, the expression of peptide Lv was detected in the outer nuclear layer (ONL), inner nuclear layer (INL), and ganglion cell layer (GCL). The scale bars represent 1.0 mm (a and a’), 0.1 mm (b, c, d, e, f), and 0.02 mm (g).

We next examined the distribution of peptide Lv mRNA in various organs, several brain regions, and the retina using RT-PCR and *in situ* hybridization (Fig. 3). The mRNA of peptide Lv was detected in the liver, lung, spleen, cerebral cortex (cortex), cerebellum, olfactory bulb, hippocampus, eyes, and intestine, with relatively higher levels in the spleen, cerebellum, olfactory bulb, and hippocampus (Fig. 3A). In various brain areas, peptide Lv mRNA was present in the cerebellum (C; Fig. 3B-a, b, c) and located mostly in the Purkinje (P) and granule (G) cell layers. In the olfactory bulb (OB; Fig. 3B-a, d), the mRNA of peptide Lv was detected in the glomerular layer (GLM), and mitral (M) and granule (G) cell layers. In the cerebral cortex (CC; Fig. 3B-a, e), peptide Lv mRNA was widely expressed. In the hippocampus (H; Fig. 3B-a, f), peptide Lv mRNA was present in the CA1-CA3 pyramidal cell layer and the dentate gyrus (DG) granule cell layer. In the mouse retina (Fig. 3B-g), the outer nuclear layer (ONL), where the cell bodies of photoreceptors are located, had the highest expression of peptide Lv mRNA, while both inner nuclear layer (INL) and ganglion cell layer (GCL) also contained peptide Lv mRNA. Since retina photoreceptors had a relatively high density of peptide Lv mRNA, we used photoreceptors for the following bioactivity assays to investigate the functional properties of peptide Lv.

### Peptide Lv was Secreted in vitro

**Figure 4:**
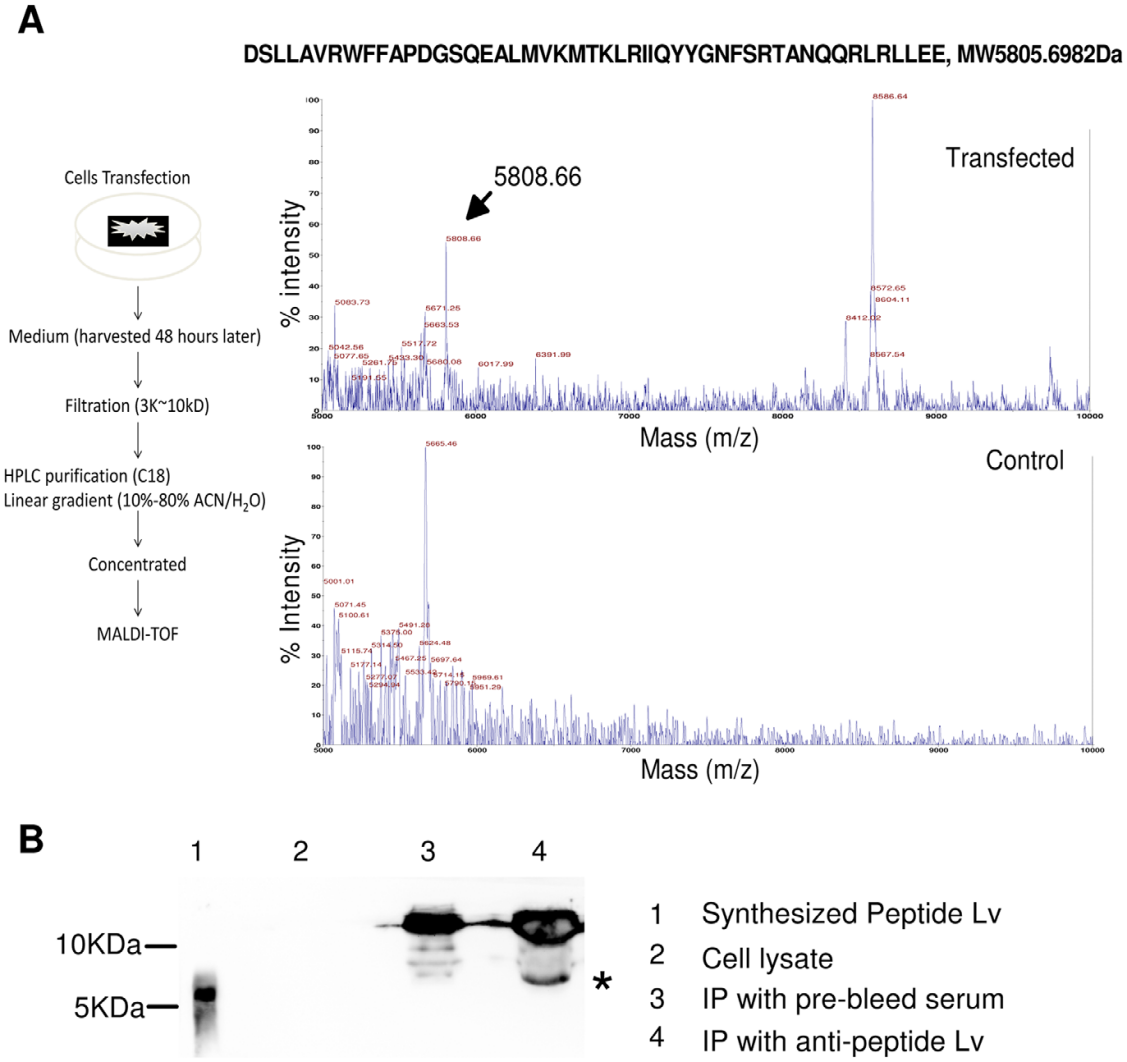
Verification of peptide Lv secretion by MALDI-TOF mass spectrometry. (A) Mouse 661w cells were transfected with the propeptide Lv gene (mouse E130203B14Rik) expression vector or empty vector (control). Culture media were collected 48 hr after transfection, filtered (3∼10 kD), and subjected to HPLC. The final elution (linear gradient 10%–80% ACN/H_2_O and 0.1% TFA in all solutions, fractions from 15–25 min retention time) was concentrated by speedvac and analyzed by MALDI-TOF MS. The arrowhead indicates the peak (5808.66 m/z) that corresponds to the predicted molecular weight of peptide Lv (5805.6982 m/z). The 49 amino acid sequence of peptide Lv is listed at the top of this panel. (B). A custom-made rabbit polyclonal anti-peptide Lv antibody against synthetic peptide Lv was obtained for detecting endogenous peptide Lv in chicken retinas. A 2 ml cell lysate sample prepared from 10 chicken retinas was used for immunoprecipitation with anti-peptide Lv antibody to concentrate (enrich) the endogenous peptide Lv in the cell lysate followed by Western immunoblotting. Lane 1 was loaded with the synthesized peptide Lv. Lane 2 was loaded with the whole retinal cell lysate (10 µl). Lane 3 was loaded with the retinal cell lysate that was immunoprecipitated with the pre-bleed rabbit serum, served as a negative control. Lane 4 was loaded with the retinal cell lysate immunoprecipitated with the anti-peptide Lv antibody. *indicates the endogenous peptide Lv pulled down from the enriched cell lysate (Lane 4).

Since one of our bioinformatic screening criteria was to find secretory bioactive peptides, we next examined whether peptide Lv was secreted from cultured cells. We transfected 661w cells, a mouse cone photoreceptor cell line, with mouse E130203B14Rik gene or an empty vector (control). If peptide Lv was expressed and secreted from transfected cells into the culture media, mass spectrometry would be able to detect the peptide from collected culture media. Transfection efficiency was monitored by coexpression of green fluorescence protein (GFP). The samples (collected culture media) were filtered through 10 kD and 3 kD cut off filters followed by partial purification through an HPLC C18 column to eliminate high molecular weight proteins, residual chemical background, and salt. The HPLC eluate was concentrated and analyzed by MALDI-TOF MS (Fig. 4). The predicted molecular weight for peptide Lv from the bioinformatic assessment was 5805.6982 D. There were five major molecular weight peaks between 5 kD and 10 kD detected by MALDI-TOF MS: 5083.73, 5671.25, 5808.66, 8412.02, and 8586.64 m/z. Compared to the molecular weights detected in the control sample (5071.45, 5375.00, 5491.28 and 5665.46 m/z), the specific molecular weight at 5808.66 m/z from the transfected samples was consistent with the predicted molecular weight for peptide Lv. Therefore, this result demonstrates that the mouse E130203B14Rik gene produced peptide Lv that was secreted from transfected 661w cells (Fig. 4). Although, there was a difference in the peaks (8586.64 m/z and 8412.02 m/z) between transfected and control samples, it was not clear whether it was a fragment derived directly from the transfected gene or other secretory fractions that was affected indirectly by the transfection of our targeted gene.

We obtained a custom-made rabbit polyclonal antibody against the synthesized peptide Lv. The antibody specifically recognized the synthesized peptide Lv (Fig. 4B, Lane 1) without cross reaction to nonspecific proteins (Fig. 4B Lane 2). Since we detected peptide Lv secretion from transfected cells, we next examined whether we could detect endogenous peptide Lv by using this antibody. Because of limitations in the amount of sample we could load, we were not able to detect peptide Lv from the whole cell lysate prepared from 10 chicken retinas (Fig. 4B, lane 2). It implies a limited or low expression or secretion of peptide Lv in the retina. We further proceeded with the immunoprecipitation by using the peptide Lv antibody to enrich the portion of endogenous peptide Lv from the whole cell lysate preparation. Since the predicted molecular weight of endogenous peptide Lv was small, we ran an 18% SDS-PAGE gel followed by Western immunoblotting with the same peptide Lv antibody. There was a specific endogenous peptide Lv positive band detected in this immunoprecipitation enriched retina sample, and the molecular weight of endogenous peptide Lv is slightly higher than the synthesized peptide Lv (Fig. 4, Lane 4). It is possible that the higher molecular weight of the endogenous peptide Lv was due to post-translational modification. The strong positive bands above 10 kDa are due to the light chains (∼27 kDa) of the antibodies. This result demonstrated the expression of endogenous peptide Lv in the retina.

### Peptide Lv Stimulated cAMP Production and ERK Phosphorylation in Chicken Cone Photoreceptors, and PTX Blocked these Effects

**Figure 5:**
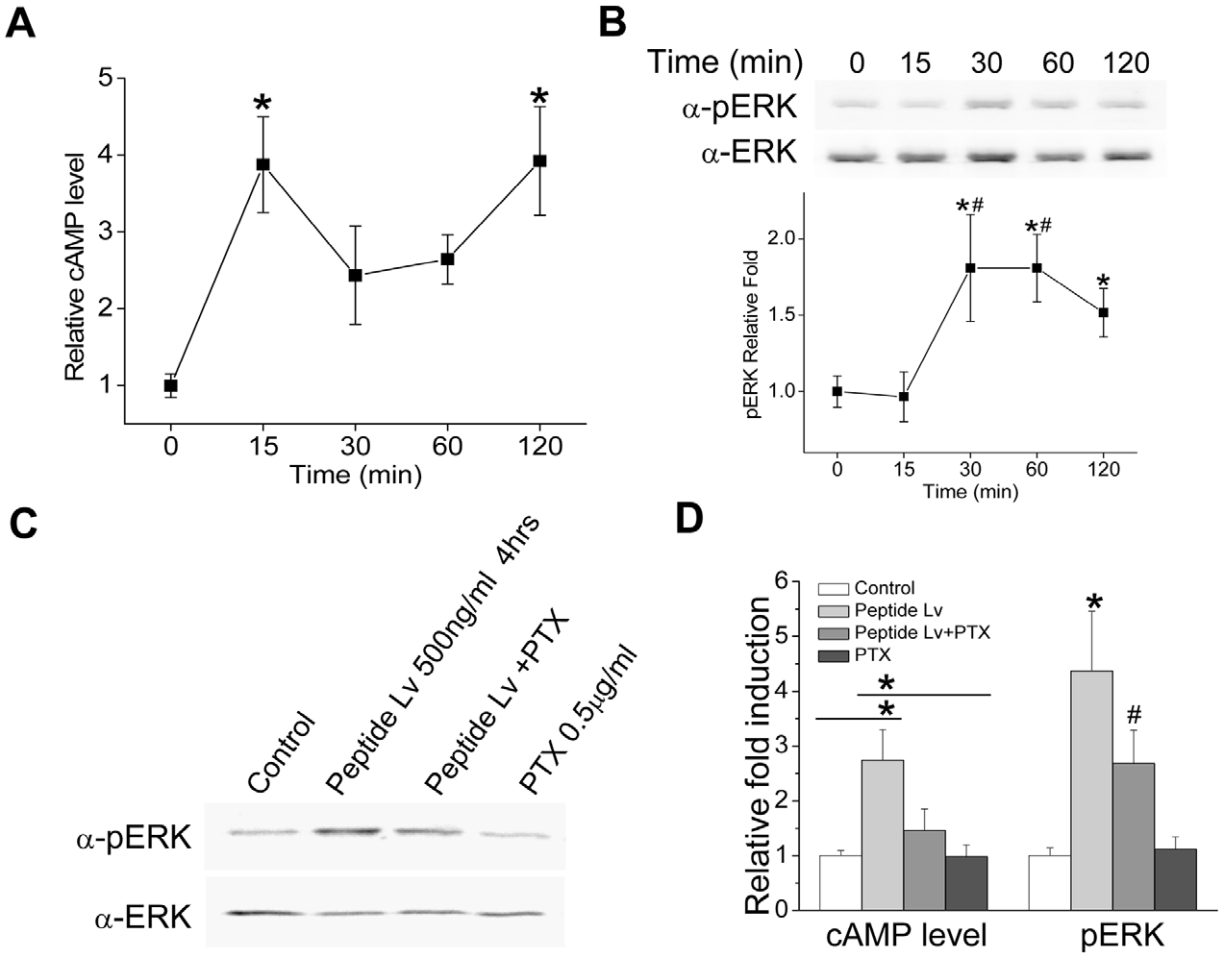
Peptide Lv enhanced cAMP production and ERK phosphorylation in chicken cone photoreceptors. (A and B) Cultured chick photoreceptors (E10+2) were treated with 500 ng/ml commercially synthesized peptide Lv or control buffer for 0, 15, 30, 60, or 120 min prior to harvest. (A) The cAMP levels increased after 15 min treatment of peptide Lv (n = 6 for each time point). *indicates that the cAMP levels at 15 and 120 min were significantly higher than 0 min (One-way ANOVA, Tukey post hoc, *p<0.05). (B) Total ERK and pERK were detected by Western blots. The band densities were measured, and the values were calculated as a ratio of pERK to total ERK. Relative fold difference was calculated by comparison to the control, in which the control was set as 1 (n = 5 for each time point). *indicates that the pERK level at 0 min is significantly different from 30, 60, and 120 min (One-way ANOVA, Tukey post hoc, *p<0.05). #indicates that pERK levels at both 30 and 60 min are significantly different from 15 min (One-way ANOVA, Tukey post hoc, ^#^ *p*<0.05). (C and D) The effect of peptide Lv on pERK and cAMP is dampened by PTX. Peptide Lv increased cAMP levels (2.74±0.56) and the phosphorylation of ERK (4.37±1.09) after 4 hours of treatment (D). However, PTX blocked the effects of peptide Lv (n = 5 for each treatment). For cAMP levels, * indicates that the peptide Lv treated group is significantly higher than the control and PTX treated groups (One-way ANOVA, Tukey post hoc, *p<0.05). For pERK levels, *indicates that the peptide Lv treated group is significantly higher than all other groups, while #indicates that the peptide Lv+PTX group is significantly higher than the control and PTX treated groups (One-way ANOVA, Tukey post hoc, both *and #indicate *p*<0.05).

Signaling by many peptide hormones or transmitters is mediated through the G-protein coupled receptor (GPCR) family [30]. Intracellular signaling pathways involving GPCRs vary depending on the coupled G-proteins and the downstream effectors [31]. However, cAMP, an intracellular second messenger, plays a key role mediating G protein signaling response to GPCRs. Therefore, we investigated whether the cAMP level changed when cells were stimulated by peptide Lv, even though we have not yet identified the specific receptor(s) for peptide Lv. We found that treatment with commercially custom synthesized peptide Lv increased intracellular cAMP levels after cells were treated for 15 min (Fig. 5A). Activation of cAMP signaling often stimulates the phosphorylation of ERK (pERK), a downstream component of the cAMP signaling pathway [32], so we next determined whether peptide Lv might also trigger phosphorylation of ERK. Treatment with synthesized peptide Lv for 30 min also elicited an increase of ERK phosphorylation (Fig. 5B). While treatment with peptide Lv for four hours elicited an increased intracellular cAMP level up to 2.74±0.57 folds (Fig. 5D) and the phosphorylation of ERK up to 4.37±1.09 folds (Fig. 5C, D), PTX diminished the peptide Lv elicited increase of cAMP and ERK phosphorylation. Treatment with PTX alone did not change cAMP levels or ERK phosphorylation compared to the control (Fig. 5C, D). Thus, PTX-sensitive G protein coupled receptors might be involved in the action of peptide Lv.

### Peptide Lv Enhanced L-VGCC Currents in Chicken Cone Photoreceptors

**Figure 6:**
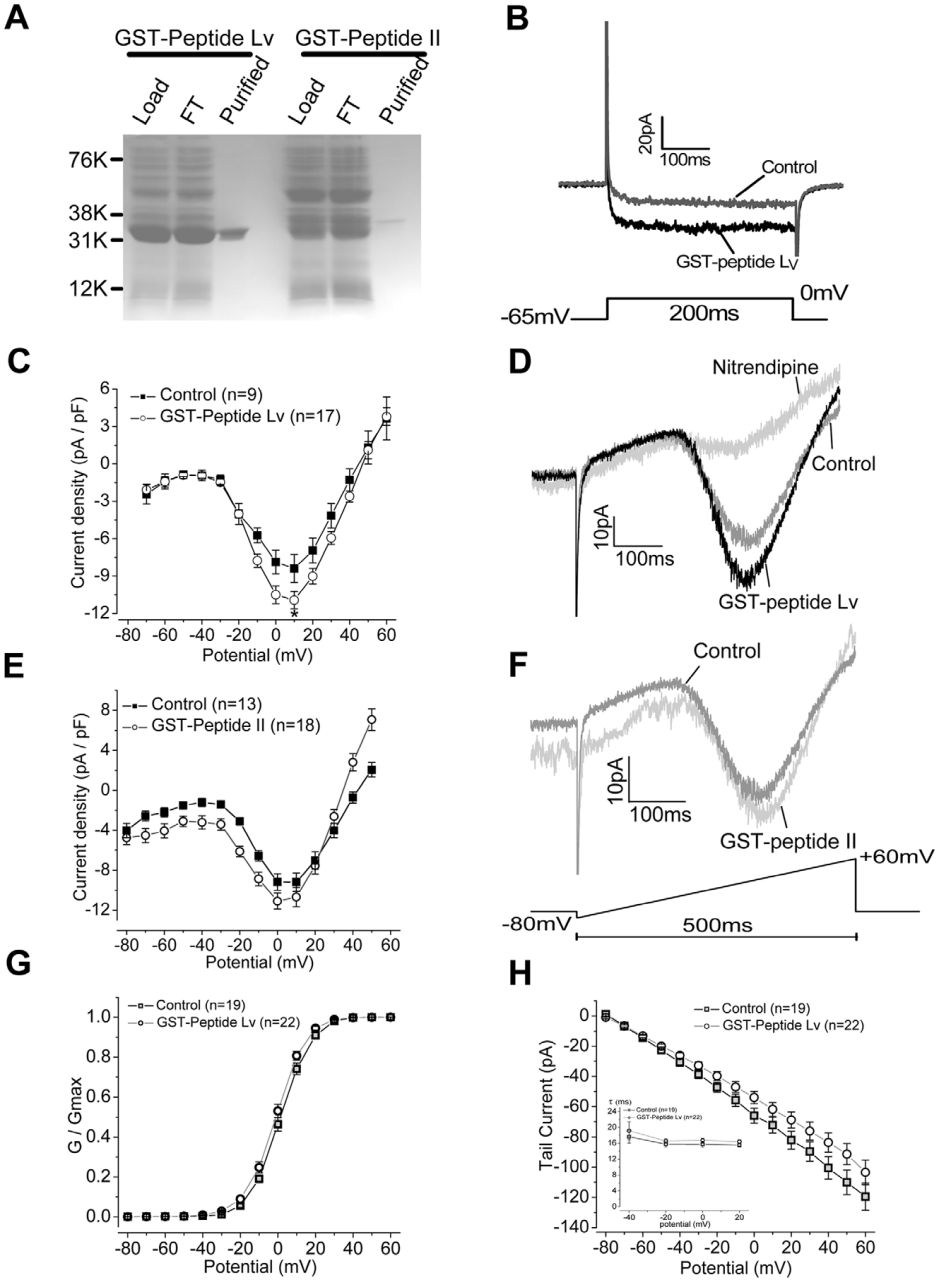
GST-peptide Lv increased L-VGCC currents in cone photoreceptors. GST-peptide Lv and GST-peptide II (control) were expressed and purified from *E. coli*. The image of lysate (Load), column flow through (FT), and the purified proteins (Purified; over 90% purity) was shown (A). (B–H) Cultured E18 (E16+2) cone photoreceptors were treated with PBS (control), 800 ng/ml GST-peptide II (control peptide), or 800 ng/ml GST-peptide Lv for 4 hrs prior to recordings. Whole cell patch-clamp recordings were performed under both step and ramp commands. Representative current traces under a step commend (B) or a ramp command (D, F) are shown. Average current-voltage (I-V) relationships were obtained from the step command as in current density (pA/pF), and the maximum inward current densities were elicited at 0 to 10 mV of the step command (C, E). (C) The average maximal current density obtained from GST-peptide Lv treated cells (n = 17, open circle) was significantly larger than the control (n = 9; filled square). Toward the end of each recording, nitrendipine (5 µM), was perfused through the extracellular solution for 5 min, and the final ramp-command was recorded. A representative current trace with nitrendipine perfusion is shown in (D). (E) There was no significant difference between the control (n = 13) and GST-peptide II treated cells (n = 18; gray circle). (G) The conductance and voltage relationship were analyzed based on cells recorded under the step command, and the G/Gmax and voltage relationship from the control (filled square) and GST-peptide Lv treated (open circle) are plotted. (H) The tail-current amplitude (pA) and Tau (τ; ms; the insert) from both control (filled square) and peptide Lv treated (open circle) were analyzed. Comparisons between the control and GST-peptide Lv or GST-peptide II treated groups were made using Student’s *t*-test; *p<0.05.

**Figure 7:**
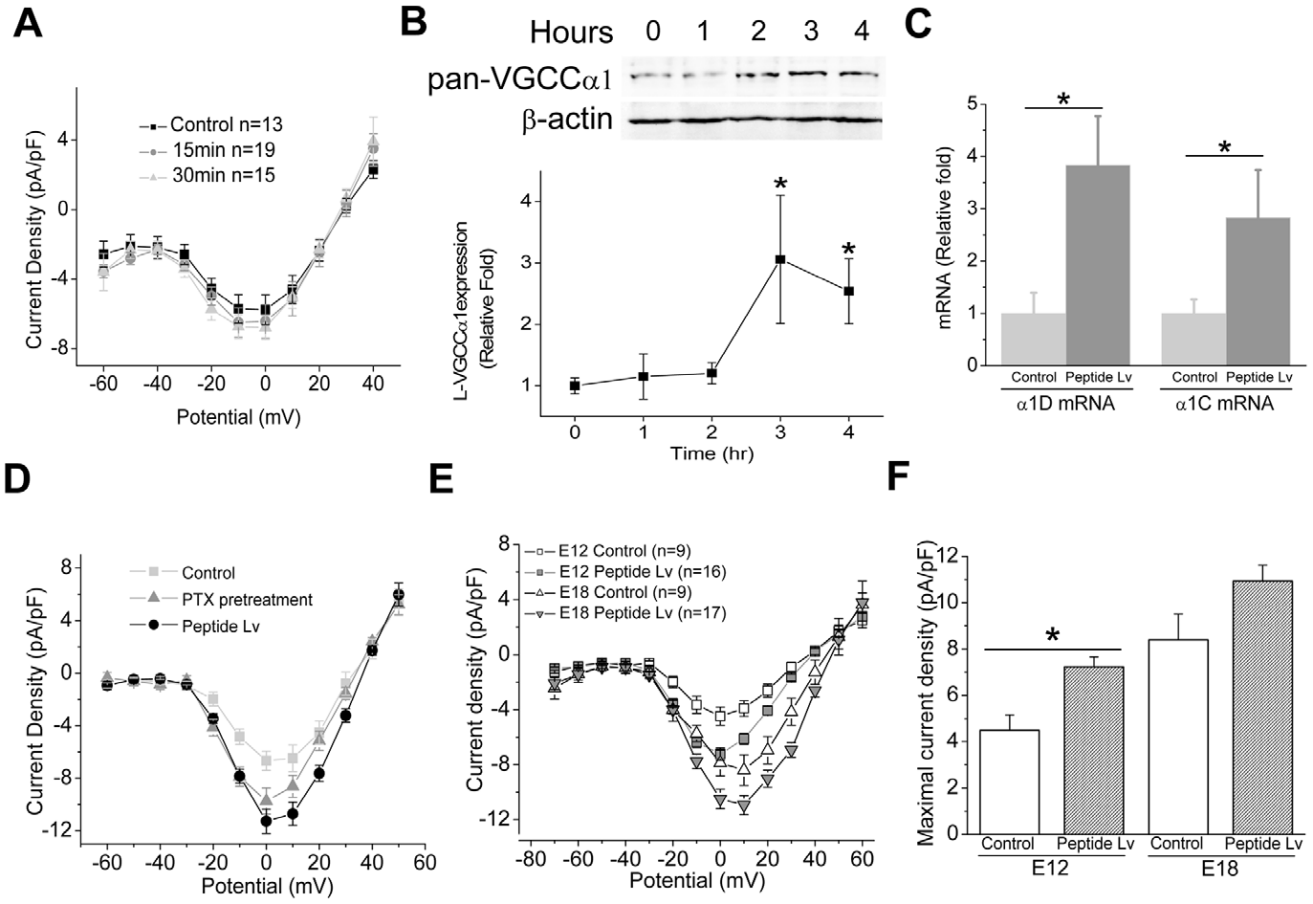
Synthesized peptide Lv increased the mRNA and protein expression of L-VGCCα1 subunit in cone photoreceptors. Cultured E18 (E16+2) photoreceptors were treated with 500 ng/ml synthetized peptide Lv for 0 (control, filled square), 15 (gray circle), or 30 min (triangle). Averaged current-voltage (I–V) relationships were obtained from the step command as in current density (pA/pF) and membrane voltage (mV). (B) Treatment with peptide Lv for at least 3 hrs elicited an increase of L-VGCCα1 subunit expression. * indicates that treatment with peptide Lv for 3 or 4 hrs is significantly different from the other groups (One-way ANOVA, Tukey post hoc, *p<0.05). (C) Treatment with peptide Lv for 4 hrs (dark gray) significantly increased the mRNA levels of both α1C and α1D compared with the control (light gray, n = 6 for each group). * indicates the statistical difference between the peptide Lv treated group and control (Student’s *t*-test; *p<0.05). (D) Compared to the treatment of peptide Lv alone (filled circle), treatment with 0.5 µg/ml PTX did not dampen the effect of peptide Lv (triangle). (E and F) The effect of peptide Lv was developmental age dependent. Chicken cone photoreceptors at E12 (E10+2) or E18 (16+2) were cultured and treated with peptide Lv. Peptide Lv significantly increased current density at E12 (E12: control, −4.49±0.66 pA/pF, n = 9, open square; peptide Lv treated cells, −7.22±0.45 pA/pF, n = 16, dark square. E18: control, −8.40±1.11 pA/pF, n = 9, open triangle; peptide Lv, −10.93±0.70 pA/pF, n = 17, dark triangle). Comparisons between the control and peptide Lv treated groups were made using Student’s *t*-test; *p<0.05.

Since cAMP and pERK have been reported to regulate L-VGCC activity in cardiomyocytes and photoreceptors [20], [33], and we showed that peptide Lv enhanced cAMP production and ERK phosphorylation (Fig. 5), we next investigated whether peptide Lv might activate L-VGCC activity in chicken cone photoreceptors. We generated a GST-fusion peptide Lv and a GST-fusion peptide II from *E. coli* (Fig. 6A) for the following electrophysiological experiments, since the GST fusion peptides can be easily expressed and purified from *E. coli*, and the recombinant peptides generated can mimic the activities of endogenous peptides [34], [35], [36], [37]. We took advantage of primary dissociated cell cultures of chicken photoreceptors for the following assays, because these cells have oil droplets present at the inner segments [22], which allow for easier identification under the microscope for patch-clamp recordings, and the cultures can be easily maintained in an incubator for several days. Cultured E18 (16+2) cone photoreceptors were treated with either GST-fusion peptide Lv, GST-fusion peptide II, or PBS (vehicle) for 4 hr prior to patch-clamp recordings. Cells treated with GST-fusion peptide Lv (800 ng/ml) had significantly higher L-VGCC current densities (10.93±0.70 pA/pF) compared to PBS treated cells (8.40±1.11 pA/pF; Fig. 6C, D), while treatment with GST-fusion peptide II did not increase L-VGCC current densities (Fig. 6E, F). Nitrendipine (5 µM), a L-VGCC blocker, abolished the L-VGCC currents in cells treated with peptide Lv (Fig. 6 D). We further obtained a commercially custom synthesized peptide Lv that had a similar purity (over 90%) to our own GST-fusion peptide Lv. There was no difference in the voltage-current relationship of L-VGCCs between treatments with either GST-fusion or synthesized peptide Lv as shown in Figs. 6C and 7D, respectively. We also analyzed the activation kinetics from the conductance-voltage (G–V) curve (Fig. 6G). While the membrane voltage of half of maximal conductance (G/Gmax at 0.5) was 1.25±1.20 mV for the control and −1.03±1.17 mV for GST-peptide Lv treated photoreceptor cells, there was no statistical difference. In the tail-current analysis, there was no difference between GST-peptide Lv treated and control cells in Tau (ms) as well as current-voltage relationship (Fig. 6H). These data imply that the increase of L-VGCC currents by GST-peptide Lv is not through changing of channel gating kinetics in photoreceptors.

Even though treatment with commercially synthesized murine peptide Lv for 15 or 30 min elicited changes in intracellular signaling, mainly increases in cAMP levels and ERK phosphorylation, treatment with commercially synthesized peptide Lv for 15 or 30 min did not alter L-VGCC currents (Fig. 7A). Therefore, the enhanced L-VGCC currents by 4 hr treatment with peptide Lv might not be en route through cAMP-dependent protein kinase A or ERK signaling for the direct phosphorylation of L-VGCC subunits in chicken cone photoreceptors. We found that treatment with peptide Lv increased the protein expression of the L-VGCCα1 subunit within 3 hr (Fig. 7B; 3.06±1.04 folds increase for 3 hr and 2.54±0.83 folds increase for 4 hr). More specifically, treatment with peptide Lv for 4 hr induced the up-regulation of both VGCCα1C and α1D mRNA levels (Fig. 7C; 2.83±0.91 and 3.83±0.94 folds, respectively). While treatment with PTX diminished the effect of peptide Lv on cAMP increase and ERK phosphorylation, PTX did not eliminate the effect of peptide Lv on L-VGCCs (Fig. 7D). Therefore, peptide Lv augmentation of photoreceptor L-VGCC currents was through increased mRNA and protein expression of the α1 subunits. While treatment with synthesized peptide Lv (500 ng/ml) on cultured E18 (16+2) photoreceptors caused an increase of L-VGCC current density, peptide Lv elicited a larger increase of L-VGCC currents on cultured E12 (E10+2) photoreceptors (Fig. 7E, F). This result demonstrates that peptide Lv had a developmental stage-dependent effect on L-VGCCs in photoreceptors.

## Discussion

In this report, we combined bioinformatic, peptidomic, biochemical, and electrophysiological methods to screen novel secretory peptides that have potential biological effects on neural physiology and function. Our *in silico* screening strategy included more features that specifically targeted secretory peptide processing, such as prohormone cleavage sites, minimal glycosylation, signal peptide motifs, and sequences conserved across different species. The purpose of this screening strategy was to provide higher numbers of potential candidates with minimal false positive selections. Our screening procedure was based on the protein database derived from full length cDNA sequences from human and mouse. As such, it is possible that some genes were not included in these databases. Additionally, we only selected candidate genes with encoded amino acid sequences that were highly conserved across different species. This criterion could rule out potential candidate genes that are species specific. We used whole-cell electrophysiological recordings on single cells as an assay method to provide a simple and quick assessment of the bioactivity of the potential candidate peptides. The sequence of peptide Lv is embedded in the mouse E130203B14Rik gene, which is known as “Mus musculus 0 day neonate eyeball cDNA, a hypothetical Immunoglobulin and major histocompatibility complex domain containing protein” (AK053685.1). In addition to peptide Lv, this gene could encode other bioactive molecules. The mRNA of peptide Lv is expressed in various organs in mice, so it is possible that its actions are not limited to the nervous system. We found that peptide Lv mRNA was heavily distributed in the photoreceptor layer of the retina. Consequently, we found that peptide Lv modulated L-VGCC activities in retinal photoreceptors, which might reveal a potential new mechanism in the regulation of neuronal function and physiology in the retina.

We found that exogenous peptide Lv enhanced calcium currents, and superfusion of L-VGCC inhibitor nitrendipine blocked the peptide Lv-augmented calcium currents, which indicates that peptide Lv indeed enhanced the L-type VGCCs, but not N- or P/Q type. It bears noting that the L-type VGCC is the major calcium channel in retinal photoreceptors [38] [18], [20]. Peptide Lv enhanced L-VGCC currents in photoreceptors may in part be through increasing the expression of mRNA and protein levels of the L-VGCCα1 subunits. However, since it took at least 3 hr of peptide Lv treatment to significantly increase mRNA and protein levels of L-VGCCα1 subunits, as well as the L-VGCC currents, we cannot exclude the possibility that our observation of mRNA and protein levels might be a consequence of other posttranscriptional and posttranslational mechanisms, which we have yet to explore. Calcium influx through the L-VGCCs is essential for neurotransmitter release in retinal photoreceptors, bipolar cells, and other non-spiking neurons [18]. Treatment with peptide Lv at E12 induced a larger increase of L-VGCC currents than at E18, indicating that peptide Lv might have a developmental stage-dependent effect or a potential trophic factor-like bioactivity. The L-VGCCs are also involved in synaptic plasticity and learning and memory [39]. In the hippocampus, long-term potentiation (LTP) is considered to be the major cellular mechanism underlying learning and memory [40]. Inactivation of *Cav1.2* (*VGCCα1C*) in the hippocampus and neocortex impairs L-VGCC dependent LTP [17]. We found that peptide Lv increased L-VGCC current density through increasing both mRNA and protein expression of L-VGCCα1 subunits after 4 hr of treatment. Interestingly, we observed high expression of peptide Lv mRNA in the mouse hippocampus. Moreover, peptide Lv elevated intracellular cAMP production and phosphorylation of ERK, both of which are critical for the induction and maintenance of LTP [41], [42]. Hence, we postulate a potential role of peptide Lv in the modulation of hippocampal LTP and synaptic plasticity. Since peptide Lv is present in different organs, it will be worthwhile to investigate additional functions of peptide Lv in the future.

The cellular signaling of many peptide hormones or transmitters is mediated through GPCRs. Over 600 GPCRs have been identified in humans and mice [30]. There are various intracellular signaling pathways that involve GPCR signaling, depending on which G-protein and downstream effector is coupled [31]. Although we have not yet identified the specific receptor(s) for peptide Lv, we found that treatment with peptide Lv increased intracellular cAMP. As a second messenger, cAMP can further activate downstream signaling components, such as Protein kinase A (PKA), RAS, and cAMP response element-binding protein (CREB), which could further regulate gene expression [32], [43]. Inhibition with PTX, a Gα-protein inhibitor, dampened the effect of peptide Lv on cAMP production and ERK phosphorylation. Interestingly, treatment with PTX did not have a significant effect on the increase of L-VGCCs by peptide Lv. Treatment with peptide Lv increased cAMP production and pERK within 15 to 30 min, but it took at least 3 hours to increase the mRNA and protein expression of L-VGCCs. In cardiomyocytes, epinephrine enhances L-VGCCs through cAMP-PKA signaling that further phosphorylate L-VGCCs [33], [44], but this pathway might not be how peptide Lv increased L-VGCCs in photoreceptors. Hence, it is possible that peptide Lv may activate different signaling pathways and cellular effects concurrently, and its actions might be cell-type specific, which will require future investigation.

In summary, we developed a new bioinformatics screening strategy, and in combination with various assay methods, we identified a novel bioactive peptide, peptide Lv. The action of peptide Lv in enhancing L-VGCCs, as well as increasing intracellular cAMP and activating ERK, indicates that peptide Lv may be important in modulating neuronal function. Identification of the specific receptor(s), signaling pathways, and other cellular activities of peptide Lv will be important future directions to investigate.

## Supporting Information

Data S1 **The Excel file contains the lists of genes in each screening step.** Sheets 1 and 2 list the human (3550 genes; sheet 1) and mouse (3164 genes; sheet 2) genes that contain potential N-terminal signal peptide sequences and secretory signals. Uncharacterized proteins (154 from both human and mouse) are listed on sheet 3, which were subjected to screening for proprotein cleavase cutting sites, transmembrane domain, glycosylation, and homology. Candidate sequences that did not have at least 50% homology among humans, rats, and mice were eliminated, which left 80 potential genes listed on sheet 4. Among these 80 candidate genes, some were further eliminated if they contained multiple transmembrane domains or signal transmembrane domains but lacked proprotein cleavase cutting motifs. The final candidate genes are listed in sheet 5.
(XLSX)
